# Patient satisfaction with pharmacy services and associated factors in Ethiopia: a systematic review and meta-analysis

**DOI:** 10.1186/s12913-025-12980-7

**Published:** 2025-07-23

**Authors:** Sisay Molla, Getachew Moges, Husien Nurahmed Toleha, Ewunetie Mekashaw Bayked, Birhanu Demeke Workneh

**Affiliations:** Department of Pharmacy, College of Medicine and Health Sciences, Wollo University, Dessie, Ethiopia

**Keywords:** Patient satisfaction, Associated factors, Influencing factors, Pharmacy services, Systematic review, Meta-analysis, Ethiopia

## Abstract

**Introduction:**

Patient satisfaction reflects the discrepancy between anticipated and actual healthcare service delivery, serving as a pivotal metric for strategic healthcare decision-making. This systematic review and meta-analysis aimed to assess the magnitude of patient satisfaction with pharmacy services and its determinants in Ethiopia.

**Methods:**

A systematic search was performed across multiple electronic databases, including PubMed, Hinari, Semantic Scholar, EMBASE, Scopus, Web of Science, and Google Scholar, to identify both published and unpublished relevant studies. Methodological quality and risk of bias were assessed using the Joanna Briggs Institute (JBI) critical appraisal tools and in accordance with the PRISMA 2020 guidelines. Statistical analyses were conducted using Stata version 17.

**Results:**

In total, 19 articles were included in the qualitative synthesis, of which 11 were selected for the quantitative analysis. The pooled prevalence of patient satisfaction with pharmacy services in Ethiopia was 56% (95% CI: 50–62), with significant associations observed with sociodemographic, socioeconomic, provider communication, and healthcare facility-related factors.

**Conclusion:**

Approximately 40% of patients expressed dissatisfaction with pharmacy services, underscoring significant systemic deficiencies. To improve healthcare quality, policymakers and healthcare administrators should prioritize the optimization of pharmacy service delivery by implementing evidence-based interventions targeting the key contributing factors identified in this study.

## Introduction

Pharmacy professionals are drug experts who provide information, optimize therapy management, promote safe and cost-effective medication use, and minimize adverse drug effects [1, 2]. Pharmacists are reliable and approachable healthcare professionals, influence healthcare policy, and are committed to delivering high-quality primary care services [2]. Good pharmacy practice (GPP) is defined as the practice of a pharmacy that responds to the needs of people who use pharmacy services by providing optimal and evidence-based care. Pharmacy professionals must prioritize patient welfare by optimizing rational, cost-effective medication use and ensuring that pharmacy objectives align with patient needs through clear communication [2–5]. All health care service providers now prioritize providing better patient care, with the ultimate goal being high patient satisfaction [6]. The healthcare sector has experienced a rapid shift in response to the growing needs and demands of its patient population, as healthcare quality is becoming an issue of worldwide concern [7]. Patient satisfaction reflects a patient’s perception of their healthcare environment, process, and outcomes. It also measures a patient’s perception of a service and the gap between their expectations and actual experience [8, 9]. Healthcare accountability is driven by patient satisfaction [10]. Patient satisfaction is a vital component and the main concern related to strategic decisions in service provisions [11]. Patient satisfaction may vary from country to country as well as from region to region owing to cultural and environmental influences and variations in the healthcare system in terms of the number and competency of healthcare providers and the organization of healthcare institutions [12]. Healthcare systems should focus on the acceptability and preferences of patients [11]. The overall prevalence of patient satisfaction with Ethiopian healthcare institutions is 63.7% [13]. However, the aim of this review was to determine patient satisfaction with pharmacy services and its influencing factors in Ethiopia.

## Method

### Search strategy

The Preferred Reporting Items for Systematic Reviews and Meta-Analyses (PRISMA) 2020 Statement guidelines were implemented in the reporting of this review [14]. A systematic literature search was conducted across multiple databases, including PubMed, Hinari, Semantic Scholar, EMBASE, Scopus, Web of Science, and Google Scholar, to identify relevant published and unpublished studies. The search strategy incorporated a combination of Medical Subject Headings (MeSH) terms and free-text keywords related to *patient satisfaction*, *pharmacy services*, and *Ethiopia*. Key search terms included: Patient satisfaction: “patient satisfaction,” “client satisfaction,” “consumer satisfaction,” “patient experience”. Pharmacy services: “pharmacy,” “pharmaceutical services,” “medication dispensing,” “community pharmacy,” “hospital pharmacy”. Geographic focus: “Ethiopia,” “Ethiopian healthcare,” “health facilities in Ethiopia”. Boolean operators (AND, OR) were used to refine the search, and filters were applied to restrict results to studies published in English without date limitations. The full search syntax for each database is available upon request to ensure reproducibility.

To minimize selection bias, reference lists of included studies were manually screened for additional relevant articles. Two independent reviewers (SM and GM) performed the search and screened studies based on predefined eligibility criteria, with discrepancies resolved through consensus or consultation with a third reviewer.

### Eligibility criteria

All relevant articles were retrieved, and the full texts were analyzed to determine which final works would be selected for this review. Numerous parameters were considered in the selection of the included studies, including the primary outcome (patient satisfaction, patient dissatisfaction), sample size (> 30), response rate (> 50%), research year (2014–2024), research design (cross-sectional, cohort study design, and case control study), and research area (Ethiopia). All papers published in English on Ethiopian patients'satisfaction with pharmacy services and associated characteristics, including those from community pharmacies and public health facilities, were included. The study period for the examined publications was restricted to 2014–2024. Studies were excluded if the data were incomplete.

### Selection process

Zotero Reference Manager Version 6 was used to eliminate redundant and unnecessary studies. Two reviewers (GM/SM) screened titles/abstracts and then independently and jointly assessed the full texts. Discrepancies were resolved via face‒to-face consensus.

### Data collection process and data items

We extracted the data via a Microsoft Excel spreadsheet. The population (study units), year of study, context, sample size, response rate, and proportions were among the outcome variables (dependent variables) that were extracted.

Qualitative data were extracted via Microsoft Word. Two reviewers (GM, SM) independently analyzed and reconciled discrepancies, consulting additional reviewers (EMB, HNT, BDM) when necessary. Missing data were obtained through author contact.

### Study risk of bias assessment

The methodological quality and risk of bias of the included studies were evaluated via the Joanna Briggs Institute (JBI) critical appraisal tools, which are tailored to specific study designs (cross-sectional) [15]. Two reviewers independently assessed each study, with discrepancies resolved through discussion or consultation with a third reviewer. Bias was evaluated via sample inclusion criteria, study subject and setting descriptions, exposure validity and reliability measurements, objective-related measurements, confounding factor identification and mitigation techniques, suitability of outcome measures, and appropriate statistical analysis. The studies were evaluated via 8 items in the JBI critical appraisal technique. Bias was categorized as low risk (studies fulfilling ≥ 80% of the JBI criteria), medium risk (studies meeting 50–79% of the criteria), or high risk (studies scoring < 50% of the criteria). The review included only low- and medium-risk studies.

### Effect measures

For all the studies, the prevalence, inverse variance, and proportions were calculated. The summary effect was estimated via the X^2^ value, z value, and *p* value with a 95% confidence interval.

### Data analysis methods

For quantitative analysis, the data (events, nonevents, participants, and sample size) were extracted via a Microsoft Excel spreadsheet. Preliminary outcome measures, such as the prevalence rate and proportion, were subsequently computed via Microsoft Excel. Finally, STATA was used to assess the overall effect sizes through a general inverse variance analysis. A random effects meta-analysis model was chosen in advance to account for expected heterogeneity across studies due to differences in populations, interventions, outcomes, or settings. The random effects model includes both within- and between-study variance, offering a more conservative and generalizable estimate, and the summary odds ratio with a 95% confidence interval was calculated.

For the qualitative synthesis, thematic analysis was employed. Thematic analysis is a qualitative method that identifies, organizes, and interprets patterns (themes) within data to uncover insights into participants'experiences, perspectives, or behaviors. It involves coding data, grouping codes into themes, and refining them to address the research question.

### Publication bias assessment

The assessment of reporting bias was conducted by considering the publication status of the studies. The year of the studies and the years in which they were published were also taken into consideration. We contacted the study authors for missing or incomplete data.

### Heterogeneity analysis

The between-study heterogeneity was assessed via the I^2^ statistic. Using inverse variance (% of weight), the impact of each study on the total meta-analysis was calculated. A funnel plot was used to investigate the potential for publication bias or bias among studies.

## Results

### Study selection

**Fig. 1:**
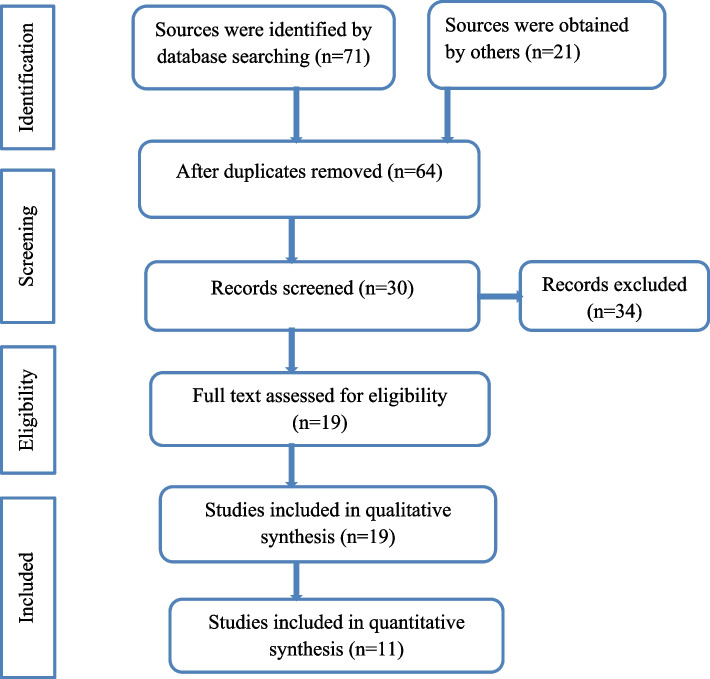
PRISMA flow diagram showing the selection process of included studies

A total of 92 sources were identified. We searched different databases (Table 1). Sixty-four records were discovered after irrelevant records were eliminated. Thirty records were checked for title and abstract review after 34 duplicated records were excluded. Nineteen records were chosen for full-text evaluation after the title and abstract were reviewed. A total of 19 studies were included in the qualitative synthesis, and 11 studies were included in the quantitative synthesis of this review (Fig. 1).

**Table 1:** Distribution of studies by database

Database	Number of studies (n)
Google Scholar	20
PubMed	19
Semantic Scholar	10
EMBASE	9
Scopus	8
Web of Science	10
Manual Google Search	16

### Study characteristics

The Amhara (*n* = 7), Oromia (*n* = 2), Afar (*n* = 1), Harar (*n* = 2), and SNNPR (*n* = 3) regions accounted for roughly (*n* = 15) 78.9% of the total studies included in the systematic review. Studies are limited in regions such as Oromia and Afar. This may be due to a lack of interest from researchers. The other studies were conducted in Addis Ababa (*n* = 4). The total sample of the population of all included studies was 7945, of which 7748 (97.5%) were found to be actual participants (Table 2).

**Table 2:** Characteristics of the individuals included in the studies, Ethiopia (*n* = 19)

Author, publication year	Year	Area	SS	RR	Study design	Main outcome	Quality score
Surur et al. 2015 [5]	2013	Amhara	405	400	cross sectional	patient satisfaction	87.5
Eshetie et al. 2020 [15]	2016	Amhara	413	408	cross sectional	patient satisfaction	100
Ayalew et al. 2017 [16]	2015	Amhara	287	287	cross sectional	patient satisfaction	75
Belete et al. 2023 [17]	2022	Amhara	308	308	cross sectional	patient satisfaction	100
Yehualaw et al. 2022 [18]	2021	Amhara	410	384	cross sectional	patient satisfaction	87.5
Molla et al. 2022 [19]	2021	Amhara	422	401	cross sectional	patient satisfaction	75
Kebede et al. 2021 [20]	2020	Amhara	422	414	cross sectional	patient satisfaction	87.5
Gidey et al. 2021 [12]	2020	Afar	422	407	cross sectional	patient satisfaction	87.5
Semegn and Alemkere 2019 [21]	2019	Adisabeba	250	245	cross sectional	patient satisfaction	100
Habte et al. 2023 [22]	2022	Adisabeba	417	407	cross sectional	patient satisfaction	75
Goben et al. 2020 [23]	2019	Adisabeba	600	589	cross sectional	patient satisfaction	87.5
Berehe et al. 2018 [24]	2016	Adisabeba	423	420	cross sectional	patient satisfaction	87.5
Ayele et al. 2020 [25]	2018	Harar	422	407	cross sectional	patient satisfaction	100
Nigussie and Edessa 2018 [26]	2016	Harar	844	844	cross sectional	patient dissatisfaction	87.5
Fekadu et al. 2020 [27]	2019	Oromia	200	195	cross sectional	patient satisfaction	75
Berhanu et al. 2022 [28]	2020	Oromia	488	439	cross sectional	patient satisfaction	75
Gamosagaro 2015 [29]	2015	SNNPR	421	415	cross sectional	patient satisfaction	75
Worku and Loha 2017 [30]	2014	SNNPR	407	394	cross sectional	patient satisfaction	75
Teshome and Kefale 2016 [1]	2016	SNNPR	384	384	cross sectional	patient satisfaction	75

*SS* sample size, *RR* response rate, *SNNPR* South Nation Nationalities People Region

### Risk of bias in studies

Studies were assessed via the Joanna Briggs Institute (JBI) criteria, with cross-sectional studies scoring 8 items: ≥ 7 (low risk), 5–6 (medium risk), and ≤ 4 (high risk) (Tables 2 and 3).

**Table 3:** Assessment results of the risk of bias for each included study

Authors	Score		Risk
	Tally	Percent (%)	
Ayele et al. [25]	8/8	100	Low risk
Gidey et al. [12]	7/8	87.5	Low risk
Habte et al. [22]	6/8	75	Medium risk
Kebede et al. [20]	7/8	87.5	Low risk
Molla et al. [19]	6/8	75	Medium risk
Semegn and Alemkere [21]	8/8	100	Low risk
Yehualaw et al. [18]	7/8	87.5	Low risk
Ayalew et al. [16]	6/8	75	Medium risk
Belete et al. [17]	8.8	100	Low risk
Teshome and Kefale [1]	6/8	75	Medium risk
Fekadu et al. [27]	6/8	75	Medium risk
Goben et al. [23]	7/8	87.5	Low risk
Eshetie et al. [15]	8/8	100	Low risk
Nigussie and Edessa [26]	7/8	87.5	Low risk
Berehe et al. [24]	7/8	87.5	Low risk
Work and Loha [30]	6/8	75	Medium risk
Gamosagaro [29]	6/8	75	Medium risk
Berhanu et al. [28]	6/7	75	Medium risk
Surur et al. [5]	7/8	87.5	Low risk

### Patient satisfaction with pharmacy services

**Fig. 2:**
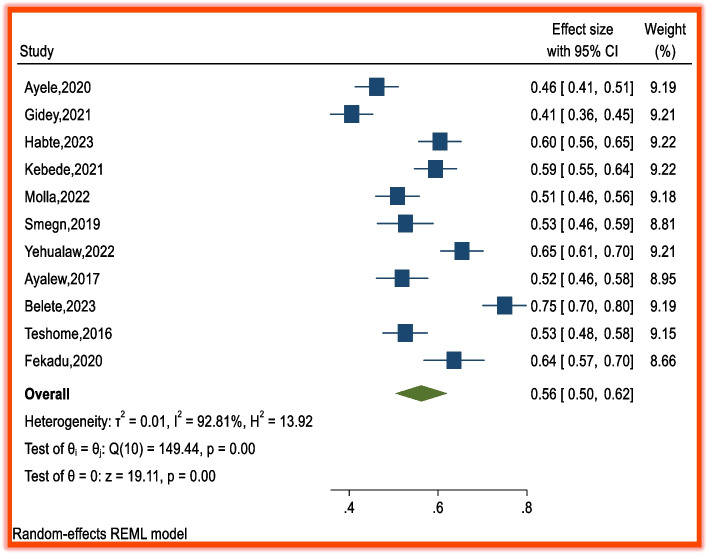
Forest plot of the pooled effects of overall patient satisfaction with pharmacy services in Ethiopia from 2014–2024

Among the included studies, 9 focused on patient satisfaction with pharmacy services among public health facilities [1, 12, 16, 18–21, 25], and the remaining studies focused on ARV patient satisfaction with services provided by pharmacists in Dembia [17], type 2 diabetes mellitus patient satisfaction with pharmacy services at Wollega University referral hospital [27], and patient satisfaction with Red Cross pharmacy services at Adis Ababa [22]. The prevalence of patient satisfaction with pharmacy services in Ethiopia ranges from 41% [12] to 75% [17]. The pooled estimate of overall patient satisfaction with pharmacy services in Ethiopia was 56% (95% CI: 50, 62), indicating moderate performance. Compared with global standards for patient-centered care, this reflects a need for improvement, especially patient counseling, medication availability, and communication. There was high heterogeneity between studies, as evidenced by a significant heterogeneity in the chi-square statistic (Q = 149, degree of freedom = 10, *p* < 0.001) and I^2^ = 92.81%, with *p* < 0.001) (Fig. 2). The high heterogeneity in patient satisfaction levels may stem from several factors. Service delivery variations: Differences in how pharmacy services are organized and delivered (e.g., hospital vs. community pharmacies) can affect patients’ experiences. Regional disparities: Access to resources, infrastructure, and staffing may vary across regions, influencing satisfaction levels. Healthcare setting differences: Public vs. private facilities may offer different standards of care, impacting patient perceptions. Patient expectations and demographics: Cultural, educational, and socioeconomic differences can shape satisfaction differently across populations. Measurement tools: Inconsistencies in how satisfaction was assessed may also contribute to variability. Measurement tools: Inconsistencies in how satisfaction was assessed may also contribute to variability.

### Subgroup analysis

We conducted subgroup analyses on the basis of region and year of the studies to detect the source of heterogeneity. However, high heterogeneity still exists. The subgroup analysis by region revealed significant disparities in patient satisfaction across Ethiopia, with estimates ranging from 41% [12] (Afar) to 75%(Amhara) [17]. The overall pooled satisfaction was 56% (95% CI: 51–62%), but regional differences were substantial (I^2^ = 92.81%, *p* < 0.001). Between-region differences (*Q* = 4.85, *p* = 0.56) were not statistically significant, suggesting variability is driven by within-region factors (e.g., facility type, patient demographics). Amhara’s high variability (I^2^ = 96.7% for 2021–2023 studies) implies localized disparities. The weighted contributions, Amhara dominated the analysis (45.8% weight) due to multiple large studies. Afar and Harar had lower weights (9.2% each), highlighting data gaps in these regions (Table 4).

**Table 4:** Subgroup analysis of patient satisfaction with pharmacy services by region

Region	Authors	Proportion (95%, CI)	Weight
Adisababa	Habte et al. 2023 [22]	0.60 (0.56,0.65)	9.2
	Semegn and alemkere 2019 [21]	0.53 (0.46,0.58)	8.8
Sub-total		0.57 (0.49,0.64)	18.0
Afar	Gidey et al. 2021 [12]	0.41 (0.36,0.45)	9.2
Sub-total		0.41(0.36,0.45)	9.2
Amhara	Kebede et al. 2021 [20]	0.59 (0.55,0.64)	9.2
	Molla et al. 2022 [19]	0.51 (0.46,0.56)	9.2
	Yehualaw et al. 2022 [18]	0.65 (0.61,0.70)	9.2
	Ayalew et al. 2017 [16]	0.52 (0.46, 0.58)	9.0
	Belete et al. 2023 [17]	0.75 (0.70,0.80)	9.2
Sub-total		0.61(0.52,0.69)	45.8
Harar	Ayele et al. 2020 [25]	0.46 (0.41,0.51)	9.2
Sub-total		0.46(0.41,0.51)	9.2
Oromia	Fekadu et al. 2020 [27]	0.65 (0.57,0.70)	8.7
Sub-total		0.64 (0.57,0.70)	8.7
SNNPR	Teshome and Kefale 2016 [1]	0.53 (0.48,0.58)	9.2
Overall		0.56(0.51,0.62)	100.00

*CI* confidence interval

**Fig. 3:**
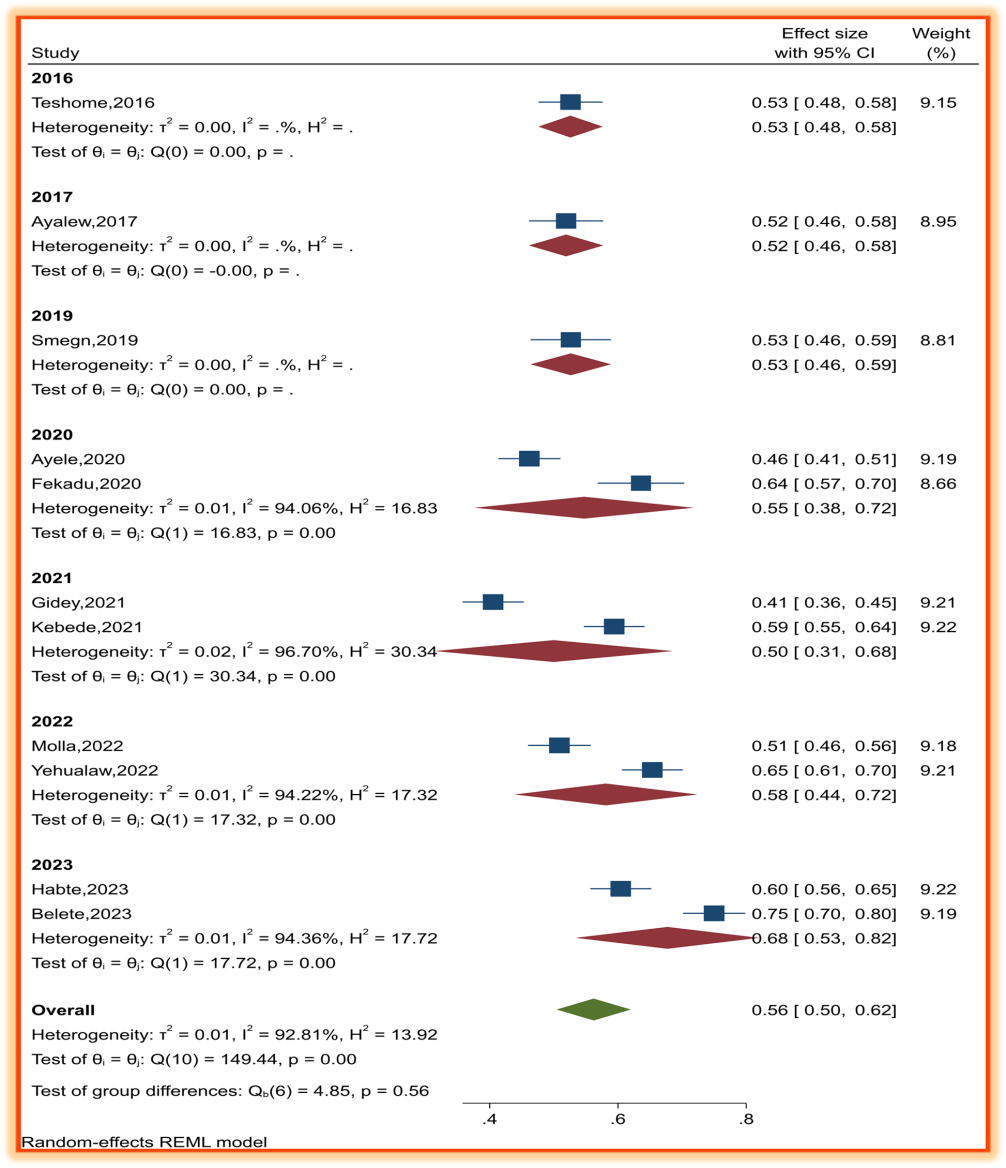
Subgroup analysis of patient satisfaction with pharmacy services by year of study

The heterogeneity of the subgroup analysis according to the years of study,2020–2023 studies showed the highest I^2^ values (94–97%), suggesting temporal or methodological shifts. Pre-2020 studies had negligible heterogeneity (I^2^ ≈ 0%), possibly due to smaller sample sizes or uniform settings (Fig. 3).

Subgroup analyses based on pharmacy ownership (private vs. public) and service area (urban vs. rural) could not be conducted to assess potential sources of heterogeneity. This limitation arose because only one study involved private pharmacies, which did not report an excessively high prevalence [22], and no studies were available for rural areas.

### Risk of bias across studies

**Fig. 4:**
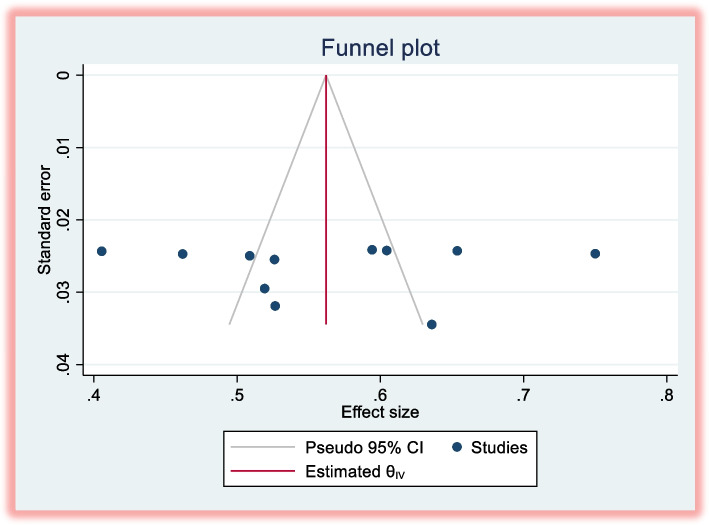
Funnel plot for publication bias in the prevalence of patient satisfaction with pharmacy services in Ethiopia, 2014–2024

The presence of publication bias was assessed via a funnel plot. The funnel plot was constructed by labeling the proportion of patients satisfied with pharmacy services (the effect size) on the x-axis and the standard error of the proportion of patients satisfied with pharmacy services on the y-axis. Funnel plot asymmetry and statistical tests (Egger’s *p* = 0.03) indicate potential small-study effects, with smaller studies overestimating satisfaction. Trim-and-fill adjustment suggests the true pooled satisfaction may be 4% lower**.** Despite bias, the original estimate (56% [95% CI: 50–62%]) remains robust, as the adjusted value (52% [47–57%]) falls within its CI. Underrepresented regions (e.g., Afar: 41% satisfaction) may skew overall estimates if their true effects are underreported (Fig. 4).

## Results of qualitative synthesis

### Associated factors of patient satisfaction with pharmacy services

#### Theme 1: Positive factors associated with patient satisfaction with pharmacy services

1. Sociodemographic factors: *Age*: older adults [5]; occupation: no job [5]*; educational status*: illiterate [1, 5]; primary and secondary educational levels [1]; residency status: rural resident [25]; *marital status*: married participant [28].
2. Socioeconomic factors: *Insurance*: insured paying service [5, 12]; *frequency of visit*: frequent one [20]; and waiting time [16].
3. Pharmacy professional communication factors include pharmacists’ commitment to correct myths [18], language pharmacists’ use [16], the professional ethics of pharmacists [16, 28], the provision of proper information about how to use medication [16, 27], proper answers to questions and doubts raised by patients [16], privacy during consultation [28, 29] and good interaction with their pharmacists [26].
4. *Health facility-related* factors: Comfort waiting area [20], pharmacist availability [18], good cleanliness [18, 29] and availability of medications [16, 27].

#### Theme 2: Negative factors associated with patient satisfaction with pharmacy services

1. *Sociodemographic factors*: *Age:* young adults [17, 20, 25]; *educational status*: above the college level [17, 25]; marital status: divorced marital status [26]; occupation: government employee [1]:
2. *Socioeconomic factor*: high medication price [12, 22] and service payer [1]
3. *Pharmacy professional communication factor*: information given by the pharmacist about the possible side effects of medications [5, 16, 27] and poor communication between clients and pharmacy professionals [24]
4. *Health facility-related* factors include inadequate and inconvenient counseling areas [16, 18, 19, 22], the unavailability of medications [12, 15, 19, 20, 22–24, 28–30], a disorganized pharmacy workflow [12, 22, 26, 28], the number of drugs dispensed [18], physical restrictions [18], maintaining a dosage regime [18], an insufficient number of pharmacy staff [21], inadequate and inconvenient waiting areas [19, 21, 31], inadequate furniture, such as chairs [31], patients’ perceived insufficient knowledge of pharmacists [26], a lack of quality system [26], inappropriate pharmacy premises [28], a pharmaceutical supply system [28], a lack of clean toilets in nearby waiting areas [24] and pharmacy locations [22].

## Discussion

Our systematic review and meta-analysis were conducted to estimate the pooled effects of the prevalence of patient satisfaction with pharmacy services and associated factors in Ethiopia. Patient satisfaction, which is determined by comparing patients’ assessment of the treatment they receive to what they expect, arises when patients believe that their needs and expectations are satisfied by the services they receive in medical facilities [32]. Patient satisfaction frequently influences a client's decision to seek guidance and attention as well as follow a treatment plan [26]. Patient satisfaction is still frequently utilized as a crucial indicator to assess the quality of health care services because patients play roles in quality assurance, such as targets, contributors, and reformers. Increased patient satisfaction with healthcare services increases individuals'behavioral intentions, such as attending follow-up appointments and adhering to prescribed treatment plans.

This improves health outcomes and encourages recommendations of the service to others [33]. However, unsatisfied patients may behave differently and are likely to experience serious consequences, such as failure to follow treatment regimens, which results in the development of complications of disease and drug resistance.

The pooled estimate of patient satisfaction with pharmacy services was 56% (95% CI: 50, 62). The pooled effects of this review were lower than those of reviews on patient satisfaction with health care services (63.7%) [13] and patient satisfaction with clinical laboratory services (66%) [34] but similar to reviews on patient satisfaction with nursing care in Ethiopia (55.15%).

The pooled estimates of patient satisfaction with pharmacy services in the regions of Ethiopia were Oromia (63.6%), Amhara (60.6%), Addis Ababa (56.8%), SNNPR (56.2%), Harar (46.2%), and Afar (40.5%).

The findings of this review revealed, in terms of age, older adults were satisfied [5] but opposed in studies conducted in Malaysia [35] and Indonesia [36]. This may be due to variations in patient expectations and cultural norms. In terms of educational level, those with primary and secondary educational levels and less [1, 5] were more satisfied than those with higher education levels. One possible explanation is that educated participants have high-quality service expectations and are aware of the role of public health facilities, which is supported by studies conducted in South Korea [37] but opposed by data reported from Malaysia [35]. This difference may be due to educated participants understanding the drawbacks of public hospital pharmacy services. In terms of residents, rural residents [25] were more satisfied than urban residents were. This may be because urban residents were more experienced. With respect to marital status, married participants were more satisfied [28].

Our findings of this review indicated, with respect to insurance, insured paying services were more satisfactory [5, 12], which was supported by studies conducted in South Korea [37] and Indonesia [36]. In terms of the frequency of visits, those with frequent visits are more satisfied [20], which is supported by studies conducted in Indonesia [36], and those with waiting times and short waiting times are more satisfied [16].

This review revealed that inadequate and inconvenient counseling areas, the unavailability of medications, disorganized pharmacy workflow, the number of drugs dispensed, physical restrictions, the maintenance of a dosage regime, an insufficient number of pharmacy staff, inadequate and inconvenient waiting areas, inadequate furniture, such as chairs, patients’ perceived insufficient knowledge of pharmacists, a lack of quality systems, inappropriate pharmacy premises, pharmaceutical supply systems, a lack of clean toilets in nearby waiting areas and pharmacy locations were health facility-related factors that caused patient dissatisfaction with pharmacy services.

In addition, key factors that cause patient dissatisfaction with pharmacy services, information not given by the pharmacist about the possible side effects of medications and poor communication between clients and pharmacy professionals are professional communication-related factors. In addition, socioeconomic factors such as high medication prices and service payers were determinants of the dissatisfaction of patients with pharmacy services.

## Conclusion

Patient satisfaction has a significant effect on treatment adherence and compliance. In this systematic review and meta-analysis, patient satisfaction with pharmacy services was relatively low. This systematic review underscores the importance of multidimensional reforms to improve patient satisfaction in public healthcare systems, offering actionable strategies for both short-term implementation and sustained improvement. While the findings provide a foundation for evidence-based policymaking, several gaps in the literature warrant further investigation.

Future studies should prioritize longitudinal research to evaluate the durability of satisfaction-focused interventions, particularly in the low-resource settings underrepresented in existing studies.

Additionally, mixed-methods approaches, including qualitative inquiries into patient perspectives, could illuminate the cultural, emotional, and contextual factors influencing satisfaction metrics, which quantitative measures alone may fail to capture. Finally, comparative analyses of intervention effectiveness across diverse healthcare models (e.g., public vs. private systems) could refine contextual applicability. Addressing these gaps would strengthen the evidence base for designing equitable, patient-centered care frameworks.

## Recommendation

The governing body of the public healthcare system should implement reforms across multiple domains to increase patient satisfaction. A recent systematic review and meta-analysis highlighted several evidence-based measures that can be prioritized for both immediate action and long-term strategic implementation.

### Short-term (immediate action) recommendation

Enhance pharmacist–patient communication: Train pharmacists in active listening and cultural sensitivity via workshops. Provide quick guidance on medication side effects/adherence. Reduce Wait Times: Implementing digital queues (e.g., SMS notifications). The peak-time operating hours are extended. Improve Medication Access: Create emergency stockpiles (e.g., insulin). Offer subsidized generics/discounts for low-income patients. Leverage Technology: Deploy SMS/call reminders for refills/adherence.

### Long-term recommendations

Integrate pharmacists into primary care teams: Formalize roles in chronic disease management (e.g., diabetes, hypertension) with shared electronic health records. Advocate for prescriptive authority for pharmacists to adjust doses or renew prescriptions. Policy Reforms for Affordability: Lobby for universal drug coverage or price caps on essential medicines.

Strengthen regulations against unethical practices (e.g., over-the-counter antibiotic sales). Strengthen Health Systems: Invest in pharmacy workforce training (e.g., WHO’s Medication Without Harm initiatives). Build supply chain resilience to prevent stock outs (e.g., regional medication hubs).

## Limitations

The findings of this review should be interpreted in light of its limitations. These include potential publication bias, as studies reporting nonsignificant or negative outcomes may be underrepresented in the literature. The heterogeneity of the studies was high. This may be due to the heterogeneity of methodologies across the included studies, such as variations in patient satisfaction metrics, cultural contexts, and healthcare settings, which may limit the generalizability of the results. Only English-language papers were included.

## Data Availability

No datasets were generated or analysed during the current study.

## Abbreviations

CI: Confidence Interval
PRISMA: Preferred Reporting Items for Systematic Reviews and Meta-Analyses
SNNPR: Southern Nations, Nationalities, and Peoples Region
SS: Sample size
RR: Response rate
JBI: Joanna Briggs Institute
CMHS: College of Medicine and Health Science
